# Investigation of Mechanism of Small Peptide Application in Enhancing Laying Performance of Late-Laying Hens Through Bidirectional Liver–Gut Interactions

**DOI:** 10.3390/ani16020164

**Published:** 2026-01-06

**Authors:** Yuanyuan Li, Xiaopeng Liao, Xiaoyue Wang, Yiping Wang, Qin Liu, Lizhi Li, Dongsheng Guo, Zhen Li

**Affiliations:** 1 School of Life Sciences and Environmental Resources, Yichun University, Yichun 336000, China; 2 College of Animal Science and Technology, Northwest A&F University, Xianyang 712100, China

**Keywords:** laying hens, egg quality, small peptide, gut microbiota, hepatic gene expression

## Abstract

The primary objective of this study was to investigate the mechanism through which small peptides regulate the productive performance and egg quality of laying hens during the late-laying period. A total of 200 Lohmann Pink laying hens, aged 400 days, were randomly assigned into a control treatment (CON) and a small peptide treatment (SP) for a 120-day treating period. Productive performance, egg quality, serum antioxidant capacity, intestinal morphology, microbial community, and hepatic gene expressions were measured. Results showed that SP supplementation significantly increased eggshell strength and albumen height, while reducing the rate of abnormal eggs (*p* < 0.05). SP notably enhanced the activities of superoxide dismutase (SOD), glutathione peroxidase (GSH-Px), and hepatic lipase (*p* < 0.05). Additionally, SP supplementation significantly increased microbial α-diversity (*p* < 0.05) and elevated the relative abundances of *Ruminococcus*, *Lactobacillus*, and *Faecalibacterium* (*p* < 0.05). Hepatic transcriptomic analysis revealed that up-regulated genes in the SP treatment were primarily enriched in steroid biosynthesis, while down-regulated genes were mainly associated with the *Yersinia* infection pathway. In conclusion, small peptide supplementation efficiently improved eggshell strength and albumen height while reducing the rate of abnormal eggs by modulating the interactions between gut microbiota and hepatic gene expressions. Our findings may provide an effective option for enhancing egg quality in the late-laying period.

## Simple Summary

Improving egg quality in aging hens is crucial for sustainable farming. This study found that dietary small peptides enhanced eggshell strength and albumen quality in late-laying hens. This improvement was linked to better antioxidant status, immunity, increased beneficial gut bacteria, and altered liver gene activity related to steroid synthesis. Small peptides show promise as a dietary strategy to maintain egg quality in older hens.

## 1. Introduction

Improving the production performance and egg quality of laying hens during the late-laying period is crucial for reducing production costs and promoting sustainable development in large-scale cage farming systems. Cage-rearing systems typically impair intestinal health, reduce feed utilization efficiency, and increase the risk of higher culling rates under high stocking densities and mobility-restricted environments, which negatively affect egg production and quality [1,2]. Therefore, strategies aimed at improving nutrient absorption and overall health are essential to enhance the productive performance and egg quality of hens in the late-laying period.

Small peptides (SPs) derived from the enzymatic hydrolysis of crude proteins are readily assimilated by the majority of bacterial communities for microbial protein synthesis and proliferation, which may further improve nutritional degradation and productive performance. Interestingly, previous studies have demonstrated that the addition of small peptides improves the growth rate of growing laying hens and broilers, promotes disease resistance by enhancing leukocyte functional and pro-inflammatory cytokine and chemokine gene transcription activities, and synergistically enhances antioxidant capacities coupled with mineral supplementation [3,4,5]. However, whether small peptides work in modulating the bacterial communities of laying hens and the underlying mechanisms remain poorly understood and have yet to be fully elucidated.

Emerging evidence suggests that a mutualistic relationship exists between hepatic lipid metabolic genes and gut microbiota, which is mediated by microbial utilization of dietary lipids and microbial production of secondary metabolites that modulate systemic homeostasis and gastrointestinal functions [6,7,8]. Whether this reciprocity effectively promotes the physiological health and improved productive performance of laying hens remains unexplored.

Therefore, in the present study, small peptides were applied to laying hens to investigate their effects on and underlying regulatory mechanisms related to physiological antioxidant capacity and laying rate. We hypothesized that SP supplementation would effectively modulate bacterial communities and proliferate probiotic diversity, functionally enhance microbe-derived signals to actively trigger hepatic gene expression and lipo-metabolic enzymatic catabolism, and eventually improve the productive performance and egg quality of laying hens.

## 2. Materials and Methods

### 2.1. Animals Preparation and Experimental Design

A total of 200 400-day-old Lohmann Pink laying hens with an average laying rate of 90.5% were randomly divided into a control treatment (CON) and a small peptide supplement treatment (SP) for 120 days of treatment. Each treatment contained 10 replicates, with 10 chickens in each replicate. Layers in the CON treatment group were maintained under standard poultry management and provided with regular feedstuff, while those under the SP treatment were given feedstuff supplemented with 0.3% small peptides based on the results of previous studies [3,4,5]. The hens were limited to an ingestion of 110 g of feed per day. Therefore, the daily intake of SPs per hen was 110 g/day × 0.3% = 0.33 g/day. Over the 120-day experimental period, the total SP supplemented per hen was approximately 0.33 g/day × 120 days = 39.6 g. Simply stated, SPs were acquired through the combination of a dephenolisation process following the proteolytic hydrolysis of cottonseed protein and degraded into 4 fractions based on its molecular weights (<1000 Da, 1000–2000 Da, 2000–5000 Da, and >5000 Da) and accounted for 68.4%, 16.7%, 8.3%, and 5.6% of total peptides, respectively. The control (CON) and small peptide (SP) diets were formulated to be isonitrogenous and isocaloric. The 0.3% SPs were added at the expense of a small, inert filler (celite) in the CON diet, not by increasing the overall protein content. The diets used in the present study are shown in Table 1, and layers were limited to ingestion of 110 g per day. Productive performance and egg quality were subsequently measured.

### 2.2. Productive Performances and Egg Quality Measurement

The 120-day period was the entire experimental feeding duration (from 400 to 520 days of age). Productive performance (egg number, weight, and deformity) was recorded daily throughout this period, and the rates presented in Table 2 are averages over the entire 120 days. Egg quality parameters (e.g., albumen height and shell strength) were measured in eggs collected on the final day of the experiment (day 120). Productive performance, including egg production, egg weight, and the number of deformed eggs, was meticulously recorded for each replicate. The egg production rate, deformity rate, and average egg weight were calculated through the following equations:

Egg production rate = Daily egg production number/Number of laying hens

Deformity rate = Daily number of deformed eggs/Daily egg production number

Average egg weight = Daily egg weight/Daily egg production number

Egg qualities, including egg shape index, Haugh unit, albumen height, eggshell thickness, and strength, were assessed at the end of the trial. Ten eggs from each replicate were selected for egg quality measurements using an egg quality analyzer (05-UM-01, Nanjing Ouxi Science and Trade Co., Ltd., Nanjing, China).

### 2.3. Serum Antioxidant Capacity, Immune Globulin, and Lipid Metabolism-Related Parameters

From each of the 10 replicates per treatment, 3 hens were sampled (total 30 hens/treatment), and their serum was pooled, but the statistical unit remained the replicate mean. A 12 h fasting treatment was first conducted before 5 mL of blood was harvested from each selected bird. Serum was separated through a 30 min coagulation at room temperature and a 10 min, 3000 g centrifugation. Antioxidant parameters including superoxide dismutase (SOD), glutathione peroxidase (GSH-px), and malondialdehyde (MDA) were determined by kis detection methods. Concentrations of serum IgA and IgG and the activities of hepatic lipase and serum lipoprotein lipase enzymes were assayed using ELISA quantitation kits. All the above indicators were measured by the Second Affiliated Hospital of Kunming Medical University, and the test kits used were provided by Nanjing Jiancheng Institute of Bioengineering (Nanjing, China).

### 2.4. Intestinal Morphological and Microbial Determination

Paraffin section of jejunum and ileum samples was conducted to determine the morphological discrepancies between CON and SP treatments. Villus height and crypt depth of both jejunum and ileum were measured randomly, and the ratio of villus height to crypt depth was calculated to determine the potential absorptivity.

Cecal contents were simultaneously collected, accompanied with the jejunum and ileum from one bird per replication on the last day of trial, which were subsequently quick frozen using liquid nitrogen. DNA from each sample was extracted for the amplification of 16S rRNA sequencing using the primer pairs of 515F and 806R (F: GTGCCAGCMGCCGCGGTAA and R: GGACTACVSGGGTATCTAAT) [9]. DNA samples with a bright main strip between 400 and 450 bp were chosen for sequencing libraries construction using TruSeq^®^ DNA PCR-Free Sample Preparation Kit (Illumina Inc., San Diego, CA USA), while a Qubit@ 2.0 Fluorometer (Thermo Scientific (China) co. Ltd., Shanghai, China) and Agilent Bioanalyzer 2100 system (Agilent Technologies, Inc. Palo Alto, CA, USA) were applied for the assessment of library quality. Sequencing data was acquired using the Illumina HiSeq 4000 platform (Illumina Inc., San Diego, CA, USA) and the sequences within an identified similarity >97% were assigned to the same operating taxonomic units (OTUs). Taxonomic annotation and the subsequent analysis of alpha diversity, beta diversity, and functional prediction were all examined based on OTU results through the Green Gene Database (https://www.re3data.org/repository/r3d100010549 (accessed on 15 June 2025)).

Alpha diversity indexes, which include the Chao1, Shannon, Simpson, and ACE indexes, were applied in analyzing the complexity of species diversity. Beta diversity, which functionally evaluates the differences in species complexity among treatments, were calculated by QIIME (Version 2.0) [10] and displayed using R software (Version 3.15.3, R Core Team, Vienna, Austria). Principal coordinate analysis (PCoA) analysis was constructed using the WGCNA package, stat packages, and the ggplot2 package in R software.

### 2.5. Liver Acquisition and Hepatic Gene Expression Measurement

Liver tissues from each bird were acquired and immediately frozen in liquid nitrogen. RNAs from each sample were isolated using an RNA kit (Takara, Dalian, China) according to the manufacturer’s recommendations and purified by Agencourt^®^ RNAClean™ XP (Beckman Coulter, Inc. Indianapolis, IN, USA). The Illumina NovaSeq 6000 platform (Illumina Inc., San Diego, CA, USA) was further applied for RNA-seq, and DESeq2 (version 1.42.0) software was used to complete differentially expressed gene statistics. Genes with an adjusted *p*-value < 0.05 (*p*. adj < 0.05) and a fold change (FC) ≥ 2.0 were included for further Gene ontology (GO) and Kyoto Encyclopedia of Genes and Genomes (KEGG) pathway enrichment analyses.

### 2.6. Functional and Gene Set Enrichment Analysis of DEGs

Gene ontology (GO) enrichment analysis of the differentially expressed genes (DEGs) was implemented by the clusterProfiler package based on Wallenius noncentral hypergeometric distribution, which can adjust for gene length bias in DEGs [11]. The KOBAS database and clusterProfiler software 4.0 were used to test the statistical enrichment of differentially expressed genes in KEGG pathways [12].

### 2.7. Statistical Analysis and Mapping

Differential analyses on productive performances, parameters, serum antioxidant related parameters, immunology, and lipid metabolism-related enzymatic activities were verified through a normal distribution test using the SAS (Version 9.3) (SAS Institute, Inc., Cary, NC, USA) procedure “proc univariate data = test normal” and subsequently underwent differential analysis through Student’s *t*-test. Results were presented as mean ± SEM. A *p*-value < 0.05 was considered to be significant, and 0.05 ≤ *p* < 0.10 was considered as a tendency.

## 3. Results

### 3.1. Effects of Supplement of Small Peptide on the Productive and Egg Quality

The productive performance and egg quality are first measured and results are shown in Table 2. Simply put, the egg production rate increased while the abnormal egg rate decreased in the SP treatment compared with the CON; however, this was not significant (*p* > 0.05). Egg quality indexes including egg weight, eggshell strength, thickness, albumen height, and Hangh unit are measured. Results in Table 1 showed that SP treatment significantly promoted eggshell strength and albumen height (*p* < 0.05). No significant discrepancies were observed in egg weight, eggshell thickness, and Hangh unit between the SP and CON treatments (*p* > 0.05).

### 3.2. Effects of Supplementation of Small Peptides on Immunity, Antioxidant Capacities and Lipo-Metabolic Parameters

Effects of supplementing small peptides on immunity, antioxidant capacities, and lipid metabolism-related parameters of Lohmann Pink laying hens were measured and results are shown in Table 3.

Physiological immunity results showed that IgA and IgG content significantly increased after supplementation of SPs compared with CON (*p* < 0.05). Antioxidant-related parameters showed that SP treatment significantly improved the SOD and GSH content (*p* < 0.05). However, the TNF-α and T-AOC contents showed no discrepancies (*p* > 0.05). Hepatic lipase activity significantly increased after receiving the SP treatment (*p* < 0.05).

### 3.3. Effects of Supplement of Small Peptide on Intestine Morphological Parameters

**Figure 1:**
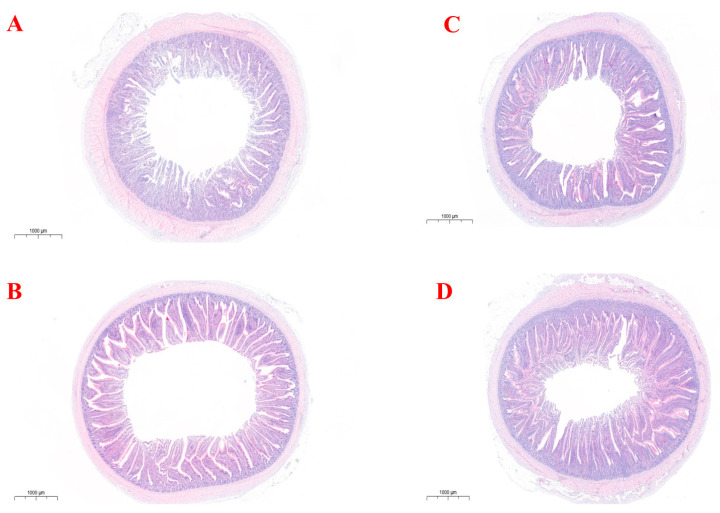
Morphological responses of the jejunum and ileum to the supplementation of small peptides in Lohmann Pink laying hens. (**A**) Morphological sections of the jejunum under CON treatment. (**B**) Morphological sections of the jejunum under SP treatment. (**C**) Morphological sections of the ileum under CON treatment. (**D**) Morphological sections of the ileum under SP treatment.

Morphological results of both jejunum and ileum section between SP- and CON-treated laying hens are shown in Figure 1 and Table 4. Villus height, crypt depth, and the V/C ratio of the jejunum and ileum showed no significant differences between the SP- and CON-treatment laying hens (*p* > 0.05).

### 3.4. Effects of Supplement of Small Peptide on Alpha Diversity

Cecal microbial responses to the SP treatment were measured, and the results are shown as follows. Holistically, a total of 8200 OTUs, 11 phyla, and more than 260 genera were identified after filtering. All taxonomic results are displayed in Table S1. The following analyses were conducted based on the OTUs.

The α-diversity of both SP- and CON-treated laying hens was first measured, and the results are shown in Table 5. Supplementation of SP showed a significant increase in the Shannon index (*p* < 0.05), and an increased tendency in the Simpson index (0.05 < *p* < 0.10). No significant alterations were found in the other indexes.

### 3.5. Effects of Supplement of Small Peptide on Beta Diversity

**Figure 2:**
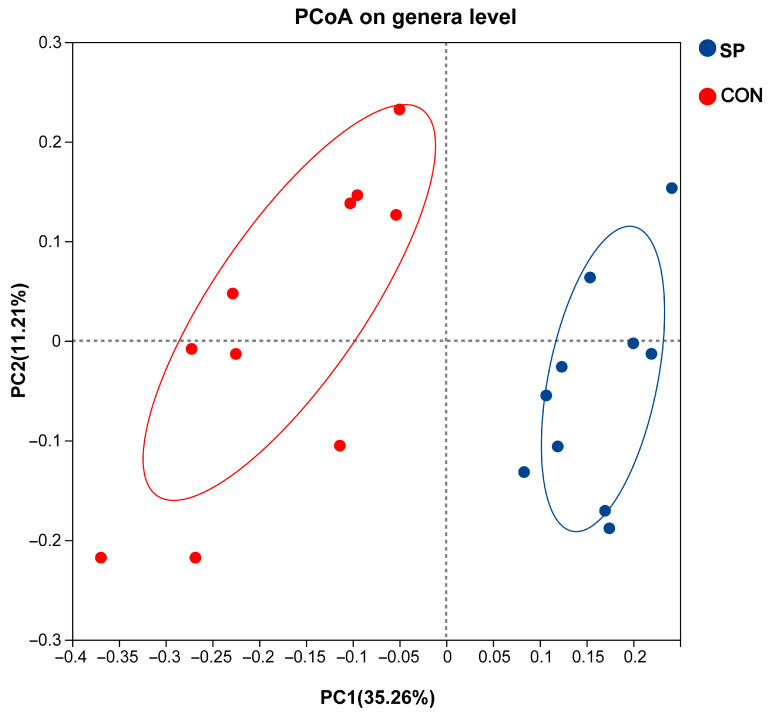
Principal coordinate analysis (PCoA) on community structures between CON and SP treatments.

Alterations in microbial communities between the SP and CON treatments were further detected using PCoA. As Figure 2 shows, PCoA axes 1 and 2 accounted for 35.26% and 11.21% of the total microbial communities. Microbial communities were significantly different between the SP and CON treatments.

### 3.6. Effects of Supplementation of Small Peptides on Gut Microbial Communities

Differential analysis on the relative abundances of cecal bacteria was further conducted between the SP and CON treatments, and results are shown in Table 6. *Bacteroides*, *Bacteroidales*, *Faecalibacterium*, *Phascolarctobacterium*, and *Ruminococcus* accounted for the top five most abundant genera. Relative abundances of Bacteroides significantly decreased (*p* < 0.05) while *Ruminococcus*, *Lactobacillus*, and *Faecalibacterium* significantly increased (*p* < 0.05) after receiving SP treatment. No significant alterations were found for other bacterial communities between the SP and CON treatments.

**Figure 3:**
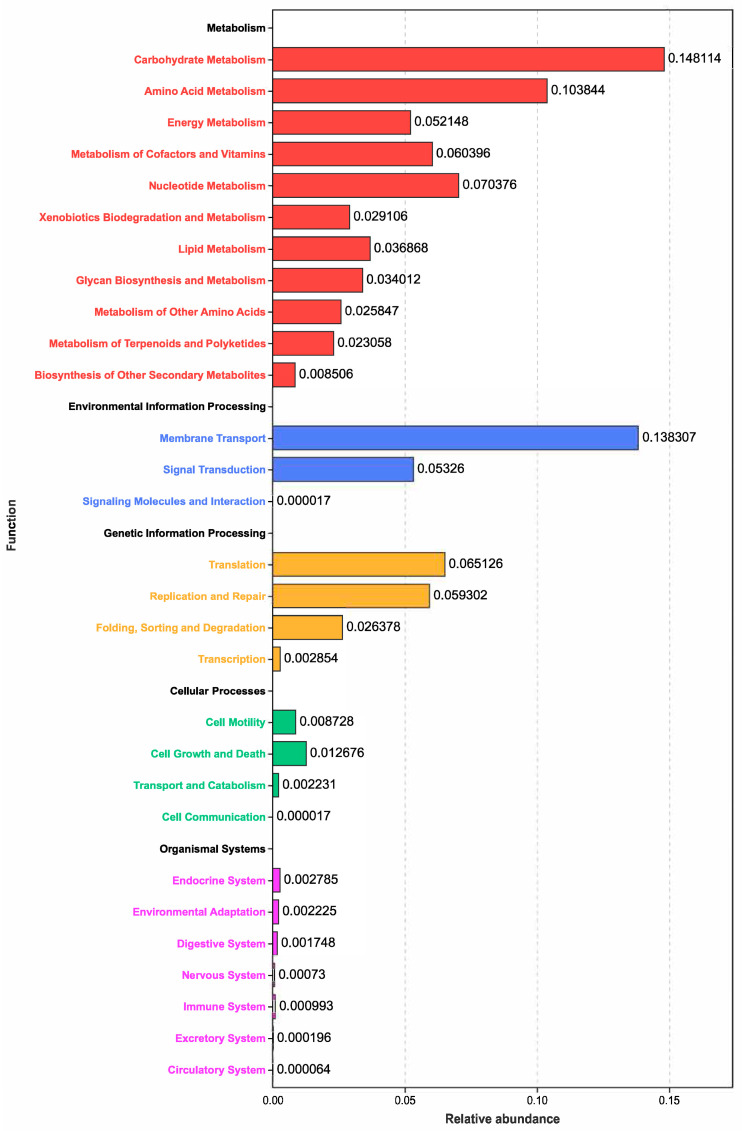
Tax4Fun2 functional prediction analysis on the differentially proliferated bacterial com-munities between the CON and SP treatments.

Predictive functions based on the differentially identified microbiota were conducted, and the results are shown in Figure 3. Metabolic processes, which mainly included carbohydrate metabolism, amino acid metabolism, energy metabolism, and lipid metabolism, were the predominant functional pathways. Other processes, including membrane transport, signaling transduction, and translation, were also mostly enriched based on the differential communities.

### 3.7. Effects of Supplementation of Small Peptides on Hepatic Gene Expressions

**Figure 4:**
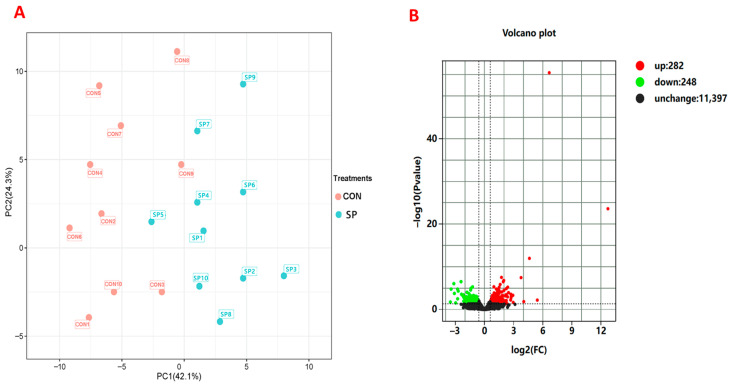
Differential analysis of the relative expressions of hepatic genes between the CON and SP treatments. (**A**) Principal component analysis (PCA) of hepatic gene expressions between the CON and SP treatments. (**B**) Volcano plot analysis of differential hepatic gene expressions between the CON and SP treatments.

Hepatic gene expression in hens from the CON and SP treatments was detected, and the results are displayed in Table S2. Holistically, a total of 11,927 mRNAs were identified after the quality filter, and all genes were used for principal component analysis (PCA). As shown in Figure 4A, PC1 and PC2 accounted for 42.1% and 24.3% of the total variation, respectively. Genes showed significantly different expression between the SP- and CON-treated birds.

Further differential expression analysis was conducted to investigate hepatic gene responses to SP supplement treatment. A volcano plot analysis was first applied for detection of significantly regulated genes. As Figure 4B shows, a total of 530 significant changed genes were detected, which included 282 up-regulated and 248 down-regulated hepatic genes, after hens received the SP supplement treatment. All the significantly altered genes are shown in Table S2.

**Figure 5:**
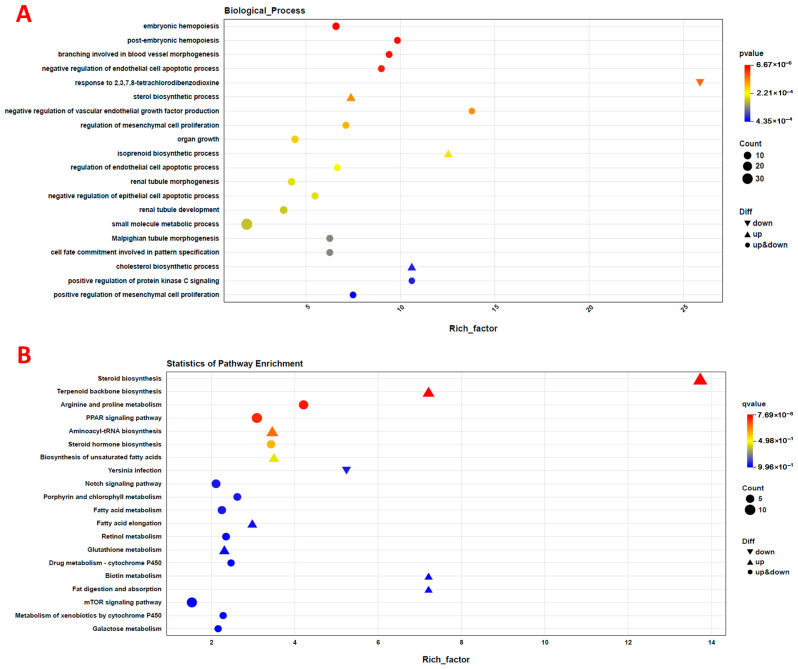
(**A**) Gene ontology (GO) and pathway enrichment analysis of the differentially expressed hepatic genes. CON and SP treatments. (**B**) Pathway enrichment analysis of the differentially expressed hepatic genes. CON and SP treatments.

All the above-mentioned differentially expressed genes are selected for functional enrichment analysis, and the results are shown in Figure 5. Figure 5A displays the GO enrichment result; the most up-regulated genes are enriched in the sterol biosynthetic process and the isoprenoid biosynthetic process, while the down-regulated genes are enriched in the response to 2,3,7,8-tetrachlorodibenzodioxine. KEGG enrichment results are shown in Figure 5B. The up-regulated genes are mainly enriched into the steroid biosynthesis, terpenoid backbone biosynthesis, and Aminoacyl-tRNA biosynthesis pathways. The down-regulated genes are mainly enriched in the Yersinia infection pathway.

### 3.8. Interactive Crosstalk Between Hepatic Genes and Cecal Bacteria on Productive Performance and Egg Quality

**Figure 6:**
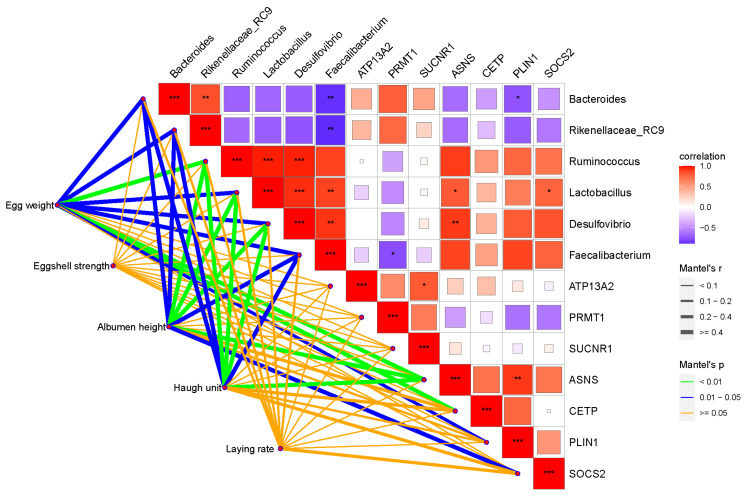
Interactive crosstalk between gut bacteria and hepatic genes on productive performance and egg quality. Note: The red blocks represent positive correlations, while the blue blocks represent negative correlations. “*” means a significant correlation (|r| > 0.55, *p* < 0.05), “**” means a significant correlation (|r| > 0.75, *p* < 0.01). “***” means a significant correlation (|r| > 0.90, *p* < 0.001). The orange lines mean no significant correlations. Blue lines indicate a significant correlation (0.01 < *p* < 0.05). Green lines indicate highly significant correlations (*p* < 0.01). ATP13A2: Polyamine-transporting ATPase 13A2; PRMAT1: Protein arginine N-methyltransferase 1; SUCNR1: Succinate receptor 1; ASNS: Asparagine synthetase; CETP: Cholesteryl ester transfer protein; PLIN1: Perilipin-1; SOCS2: Suppressor of cytokine signaling 2.

The regulatory effects of gut bacteria and hepatic genes on productive performance and egg quality were investigated and visualized in Figure 6. Laying rate and eggshell strength showed weak correlations with the significantly differentiated hepatic genes and gut microbiota. In addition, egg weight, albumen height and Haugh unit were significantly correlated with bacterial communities; only the genes of ANAS showed a significant correlation with egg quality indexes.

Specifically, *Bacteroides* and *Rikenellaceae_RC9* were positively correlated with each other and significantly negatively correlated with *Faecalibacterium*. *Ruminococcus*, *Lactobacillus*, *Desulfovibrio*, and *Faecalibacterium* were positively correlated with each other. *Bacteroides*, *Rikenellaceae_RC9*, *Ruminococcus*, *Lactobacillus*, *Desulfovibrio*, and *Faecalibacterium* were significantly correlated with egg weight, albumen height, and Haugh unit. Additionally, *Bacteroides* showed a negative correlation with PLIN1, while Lactobacillus showed positive correlations with ANAS and SOCS2. Genes of ANAS and PLIN1 were positively correlated with each other and showed significant regulatory effects on egg weight, albumen height, and Haugh unit.

## 4. Discussion

Laying rate typically declines after 400 days of age, given age-related functional decline of reproductive organs, which significantly impacts economic viability and the sustainability of the laying hen industry. Previous studies have suggested that strategies such as environmental modification, improvements in nutrient digestibility and absorption efficiency, and enhancements in physiological health can dynamically influence microbial communities [13,14,15], which dynamically interact with host gene expression and reciprocally adapt to external stimuli to maintain physiological homeostasis and sustain production performance [16]. In the present study, we explored how gut microbiota and hepatic gene expression are modulated by small peptide supplementation to investigate the underlying mechanisms influencing laying performance and egg quality in late-laying hens. Our findings may contribute to further understanding of the interplay between gut microbiota and hepatic gene expression in optimizing egg production and quality.

Physiological homeostasis serves as the functional foundation for sustaining productive performance in laying hens, with physiological antioxidant capacity and immune competence playing pivotal roles. The establishment of intestinal homeostasis provides a favorable niche for microbial colonization and gut maturation—a coordinated process dependent on host health [17]. Supplementing with SP improved systemic health, as evidenced by enhanced immune function and antioxidant capacity, with reasons for this possibly being attributed to the following aspects. SP supplementation provides highly available nitrogen resources and helps proliferate microbial α-diversity, which is shown in our findings. In addition, SP supplementation significantly elevates probiotic populations, such as the *Bifidobacterium* and *Lactobacillus* [18,19], which help increase IgA-mediated prevention of pathogenic colonization [20]. Interestingly, IgA content significantly increases after SP supplementation, which may further interact with microbial shifts in reducing pathogen abundance and strengthening intestinal barrier integrity.

Further, a synergistic interplay between hepatic gene expression and gut microbiota further modulates physiological processes. *SOCS2* is the critical negative regulator of growth hormone (GH); animals generally display gigantism with increased body weight and length when lacking in *SOCS2* expression [21]. *SOCS2* has also exhibited inhibition effects on hepatoblastoma metastasis via down-regulation of the JAK2/STAT5 signal pathway, which indicates potentially promotive impacts on physiological immunity [22,23]. Interestingly, *SOCS2* shows a positive correlation with Lactobacillus, which further proves the synergistic effect between hepatic genes and gut microbes in improving body immunity [24]. Moreover, up-regulation of energy metabolism-related genes (such as *ANAS*), positively correlated with Bifidobacterium and Lactobacillus, may further increase energy utilization, help alleviate fat deposition, and provide availability in metabolic precursors for antioxidant enzyme activation to enhance antioxidant capacity [22].

The Haugh unit is calculated using both albumen height and egg weight (HU = 100 × log (H − 1.7 × W^0.37^ + 7.6), where H is albumen height in mm and W is egg weight in grams). In our study, while albumen height increased significantly in the SP group, the concurrent but non-significant increase in average egg weight (64.35 g vs. 63.36 g) likely moderated the overall change in the Haugh unit calculation, resulting in a non-significant difference. Regarding eggshell strength and thickness, strength is a comprehensive indicator of the eggshell’s mechanical resilience, influenced not only by thickness but also by microstructure, mineral composition (such as calcite crystal arrangement), and organic matrix quality. Our findings suggest that small peptide supplementation may improve the micro-architecture or mineralization efficiency of the eggshell, enhancing its strength without necessarily altering its macroscopic thickness. Productive performance of laying hens was critically modulated by key modulatory factors, including nutritional status, physiological health, environmental stressors, and nutrient absorption efficiency [25,26]. As shown in our results, laying rates were not significantly correlated with altered microbiota communities and hepatic genes. Reasons for this might include the following aspects. Typically, laying performance is primarily modulated by key genes that encode follicular development, eggshell formation, and hormone levels. Microbiota in the cecum express assistant roles that promote efficient digestion in the intestine and feed utilization [27]. SP supplementation in our study increased cecal microbial α-diversity and probiotic abundance and partially improved nutrient degradation and absorption, which caused a slight increase in laying rate; however, this was not significant.

Albumen height significantly increased after SP supplementation, and shows a significantly positive correlation with microbial communities, including *Ruminococcus*, *Lactobacillus*, *Desulfovibrio*, and *Faecalibacterium*, and hepatic genes, including *SOCS2* and *ANAS*. The differentially expressed genes and discrepantly proliferated bacterial communities further shaped the egg quality through the following mechanism.

Increased energy provision dynamically enhanced calcium absorption and transportation to help eggshell formation [28], which may be induced by larger microbial communities and the activated hepatic genes. The increased cecal microbial α-diversity and probiotic abundance (e.g., Lactobacillus) fostered intestinal homeostasis and improved enzymatic degradation of nutrients [29,30], which subsequently caused the enhancement of epithelial absorptivity. Further, the proliferated small peptide and higher abundant microbial communities helped degrade carbohydrates into short chain fatty acids (SCFA) [31], which were systemically absorbed and activated hepatic energy metabolism genes (such as *ATP13A2*) to support eggshell formation and protein synthesis [32]. Contrarily, differentially expressed hepatic genes helped enhance antioxidant capacity and physiological immunity, as mentioned above, reducing inflammatory responses of the oviduct, and further enhanced eggshell strength and albumen height. In addition, liver-derived bile acids interacted with bile acid TGR5 (Takeda G protein-coupled receptor 5), recirculated to the proximal intestine, which functionally modulated gut microbiota composition and promoted epithelial regeneration and barrier integrity [33]. These alterations may further indirectly provide higher energy for eggshell formation and albumen protein synthesis.

## 5. Conclusions

In short, our study demonstrates that dietary supplementation with small peptides effectively enhances egg quality in late-laying hens, primarily by improving eggshell strength and albumen height. These benefits are mediated through a synergistic mechanism involving the gut–liver axis. Specifically, small peptides modulated the cecal microbiota by increasing microbial α-diversity and promoting the proliferation of beneficial genera such as *Ruminococcus*, *Lactobacillus*, and *Faecalibacterium*. Concurrently, hepatic transcriptome analysis revealed that small peptide supplementation up-regulated genes enriched in the steroid and terpenoid backbone biosynthesis pathways, while down-regulating genes associated with the *Yersinia* infection pathway. Furthermore, the supplementation enhanced systemic antioxidant capacity and immune function. The correlation analysis uncovered significant interactions between these differential gut microbes and hepatic genes, which were closely associated with key egg quality parameters. Therefore, small peptide supplementation represents a promising nutritional strategy to ameliorate age-related declines in egg quality by orchestrating bidirectional liver–gut interactions, providing a theoretical foundation for extending the laying cycle in modern poultry production.

## Figures and Tables

**Table 1:** Composition and nutrition level of the experimental diets.

Ingredient	CON	SP
Corn	61.1	61.1
SBM, CP 43%	25	24.7
Soybean oil	1.3	1.3
CaCO_3_	7.0	7.0
Calcium hydrophosphate (2 water) DCP	2.0	2.0
Salt	0.4	0.4
Small peptide	0	0.3
L- Lys-HCL, (98%)	0.1	0.1
DL-Met	0.1	0.1
Primix *	3.0	3.0
Total	100	100
ME/(MJ/kg)	11.51	11.51
CP	15.5	15.6
Ca	3.15	3.15
P	0.43	0.43
dLys	0.8	0.8
dMet	0.35	0.35
dCys	0.28	0.28
dM + C	0.63	0.63

* Vitamin content: VA 12,000 IU/kg; VD 3950 IU/kg; VE 18 IU/kg; VK 8 mg/kg; VB1 0.6 mg/kg; VB_2_ 4.8 mg/kg; VB_6_ 1.8 mg/kg; VB_12_ 10 mg/kg; Folic acid 0.15 mg/kg; niacinamide 30 mg/kg; pantothenic acid 10.5 mg/kg; choline 480 mg; Fe 80 mg; Cu 8 mg; Mn 80 mg; Zn 60 mg; Se 0.15 mg; I 0.35 mg. SBM: soya bean meal. ME: metabolizable energy. CP: crude protein. dLys: digestible lysine. dMet: digestible methionine. dCys: digestible cysteine. dM + C: digestible methionine + cysteine.

**Table 2:** Effects of supplementation of small peptides on the productive performance and egg quality of laying hens (*n* = 10).

Items	SP	CON	SE	*p*-Value
Laying rate (%)	92.47	90.38	1.71	0.064
Abnormal egg rate (%)	3.92	5.04	0.61	0.013
Egg weight (g)	64.35	63.36	4.73	0.813
Egg shape index	1.31	1.31	0.03	0.717
Eggshell strength (Kgf)	58.74	53.53	2.31	0.022
Albumen height	8.19	6.58	1.06	0.021
Haugh unit	85.14	82.75	5.02	0.755
Eggshell thickness (mm)	0.35	0.34	0.02	0.672

SP = small peptide supplement treatment. CON = control treatment. SE = standard error.

**Table 3:** Effects of supplementing small peptides on the immunity, antioxidant capacities, and lipid-metabolizing enzymes of laying hens. (*n* = 10).

Items	SP	CON	SE	*p*-Value
IgA (g/L)	2.71	2.21	0.24	0.016
IgG (g/L)	13.21	12.29	0.25	0.035
SOD (U/L)	19.17	13.06	1.28	0.027
MDA (mmol/L)	3.61	4.34	0.52	0.102
GSH (U/L)	21.22	17.86	2.23	0.024
T-AOC (mmol/L)	3.82	3.51	0.47	0.322
Hepatic lipase (U/mg)	170.4	163.4	1.73	0.010
Serum lipoprotein lipase (U/mg)	6.53	6.37	0.08	0.259

Note: SP: Small peptide supplement treatment. CON: Control treatment. SE: Standard error. The same as in the following table. SOD: Superoxide dismutase. MDA: Malonaldehyde. GSH: Glutathione peroxidase. T-AOC: Total antioxidant capacity.

**Table 4:** Effects of supplementing small peptides on the morphological parameters of the jejunum and ileum. (*n* = 10).

Items		SP	CON	SE	*p*-Value
Jejunum	Villus Height (μm)	719.1	708.2	6.75	0.298
	Crypt depth (μm)	113.2	108.1	2.79	0.136
	V/C	7.02	7.01	0.27	0.569
Ileum	Villus Height (μm)	741.1	736.3	5.76	0.378
	Crypt depth (μm)	103.2	101.4	3.79	0.416
	V/C	7.21	7.26	0.37	0.529

**Table 5:** Effects of supplementation of small peptides on the microbial α-diversity of laying hens (*n* = 10).

Index	SP	CON	SE	*p*-Value
Ace	1938.5	1891.9	106.23	0.556
Chao	1845.9	1807.4	57.72	0.462
Coverage	0.991	0.988	0.007	0.441
Shannon	5.06	4.52	0.117	0.021
Simpson	0.031	0.043	0.012	0.187
Sobs	1353.5	1336.2	26.14	0.548

**Table 6:** Effects of supplementing small peptides on the bacterial communities of laying hens (%, level of genera, *n* = 10).

Items	SP	CON	SE	*p*-Value
g__*Bacteroides*	27.12	33.32	2.75	0.042
g__*Bacteroidales*	11.29	10.42	2.72	0.711
g__*Faecalibacterium*	6.31	4.66	0.74	0.023
g__*Phascolarctobacterium*	4.78	4.92	1.15	0.864
g__*Ruminococcus*	5.07	4.81	0.67	0.512
g__*Lactobacillus*	5.54	3.13	1.08	0.024
g__*Desulfovibrio*	2.93	2.42	0.61	0.404
g__*Megamonas*	3.51	1.43	1.40	0.142
g__*Synergistes*	1.82	2.63	0.91	0.123
others	32.72	33.27	1.85	0.613

## Data Availability

The raw data supporting the conclusions of this article will be made available by the authors on request.

## Supplementary Materials

The following supporting information can be downloaded at https://www.mdpi.com/article/10.3390/ani16020164/s1: Table S1: All taxonomic results; Table S2: Hepatic gene expression in hens from the CON and SP treatments.
